# Implementation and Dosimetric Evaluation of a Knowledge‐Based IMRT Model for Left‐Sided Breast Radiotherapy with DIBH

**DOI:** 10.1002/acm2.70599

**Published:** 2026-05-28

**Authors:** Oznur Senkesen, Emine Burcin Ispir, Irem Aydin, Evrim Tezcanli

**Affiliations:** ^1^ Acıbadem Mehmet Ali Aydınlar University Istanbul Türkiye; ^2^ Department of Radiation Oncology Acıbadem Ataşehir Hospital İstanbul Türkiye; ^3^ Department of Radiation Oncology Acıbadem Altunizade Hospital İstanbul Türkiye

**Keywords:** DIBH, Halcyon, IMRT, knowledge‐based planning, left‐sided breast cancer, RapidPlan

## Abstract

**Purpose:**

This study aimed to dosimetrically evaluate the plan quality of a knowledge‐based planning (KBP) model developed from intensity‐modulated radiation therapy (IMRT) plans for left‐sided breast cancer treated with deep inspiration breath hold (DIBH), and to assess its clinical applicability.

**Materials and methods:**

Eighty patients previously treated with DIBH were included. A RapidPlan‐based KBP model was developed using clinically approved plans (ClinP) within a Halcyon‐equivalent workflow on the Ethos platform. Dosimetric performance of model‐only plans (KBP) and plans refined with limited manual optimization (KBP+MP) was compared against ClinP for planning target volume (PTV) coverage and organ‐at‐risk (OAR) sparing.

**Results:**

The KBP model demonstrated satisfactory training performance, with acceptable coefficients of determination (R^2^) and mean squared error (MSE) values for most OARs. Both KBP and KBP+MP achieved PTV coverage comparable to ClinP, particularly for D95 and D98. In the left lung, V20 and D2% were reduced, and both the mean dose and D2% to the left anterior descending (LAD) artery were significantly lower in KBP plans, although lung V5 showed a slight increase.

**Discussion:**

Our findings support the clinical applicability of KBP. While KBP effectively reduces high‐dose regions, it may increase low‐dose exposure particularly in distant OARs such as the contralateral lung and breast. The KBP+MP approach provided more balanced OAR sparing while simplifying the overall planning workflow compared with manual methods.

**Conclusion:**

The KBP model demonstrated clinical feasibility for left‐sided breast IMRT under DIBH conditions. Model‐generated plans achieved target coverage comparable to clinically approved plans while providing acceptable OAR sparing. Although KBP alone tended to increase low‐dose exposure in contralateral structures, limited manual refinement improved overall dose balance. These findings support the integration of structured KBP workflows into standardized DIBH breast IMRT planning.

## 1 INTRODUCTION

Breast cancer remains the most commonly diagnosed cancer among women worldwide.^1^, ^2^ Postsurgical radiotherapy following either mastectomy or breast‐conserving surgery is fundamental to reducing the risk of local recurrence. When regional involvement is suspected, additional irradiation of the supraclavicular and/or internal mammary lymph nodes is often indicated.^3^

The increasing use of advanced radiotherapy techniques such as intensity‐modulated radiotherapy (IMRT) and volumetric modulated arc therapy (VMAT) enables more homogeneous target coverage while better sparing adjacent organs at risk (OARs).^4^, ^5^ Delivering an effective tumor dose while minimizing exposure to surrounding tissues, particularly the heart, lungs, and left anterior descending artery (LAD) is especially important in left‐sided breast cancer.^6^, ^7^

One technique that has gained widespread adoption for this purpose is deep inspiration breath hold (DIBH). By encouraging patients to hold a deep breath during irradiation, the lungs expand and the heart is displaced farther from the treatment fields, resulting in lower doses to critical structures.^8^, ^9^, ^10^ In our clinic, left‐sided breast radiotherapy is delivered under DIBH on a Varian Ethos™ O‐ring linear accelerator integrated with a surface‐guided radiation therapy (SGRT) system and operated in nonadaptive mode using a Halcyon‐equivalent IMRT workflow.

Over the years, breast radiotherapy has advanced in parallel with improvements in both treatment delivery and planning systems. Techniques have evolved from three‐dimensional conformal radiotherapy (3D‐CRT) and forward‐planned IMRT to inverse‐planned IMRT, VMAT, and helical TomoTherapy. Despite these developments, creating high‐quality treatment plans remains demanding. Outcomes are influenced by patient anatomy, the planner's level of experience, and institution‐specific clinical goals. Studies have shown substantial inter‐planner variability even within single facilities: Nelms et al. attributed this to subjective differences in optimization strategies, while Fried et al. highlighted the strong dependence of plan quality on planner expertise.^11^, ^12^

To address these inconsistencies while maintaining high dosimetric standards, knowledge‐based planning (KBP) has been introduced as a promising approach. KBP uses historical data from previously approved plans to generate predictive models that guide the planning of new cases. By referencing dose–volume histogram (DVH) metrics from a library of prior cases, KBP can streamline workflow and improve plan consistency.^13^, ^14^ Scaggion et al. further suggested that KBP can support less‐experienced planners, promoting greater standardization in clinical practice.^15^ Additional studies have shown that KBP can reduce planning time while preserving or even enhancing overall plan quality, including in complex scenarios such as DIBH for left‐sided breast cancer.^13^, ^16^, ^17^

Although KBP has been explored in breast radiotherapy, limited data exist regarding structured IMRT‐based implementation under DIBH conditions incorporating independent validation and post‐deployment clinical assessment. The present study aims to address this gap by evaluating model performance, the impact of limited manual refinement, and workflow feasibility within a standardized clinical framework.

## 2 MATERIALS AND METHODS

### 2.1 Study cohort and design

A total of 80 patients previously treated for left‐sided breast cancer at our clinic were included. At least 20 plans are considered sufficient to create a KBP model in Eclipse.^1^ ^8^ However, variability in breast anatomy and cohort heterogeneity prompted us to initiate model development with 60 patients. After excluding outliers, 45 cases were retained for final model training. Within the overall cohort, 45 cases were used for model training, 20 for validation, and 15 for evaluating clinical usability in the post‐implementation phase. Cases identified as outliers during the model training stage were excluded only from the training dataset but remained within the overall cohort and were incorporated into the validation population to assess model robustness.

Both conventional fractionation (50 Gy in 25 fractions) and moderate hypofractionation (40 Gy in 15 fractions) regimens were included in the model to assess its generalizability across different dose schedules. Regardless of fractionation type, all plans were evaluated for target coverage and OAR sparing during both training and validation phases. The detailed patient distribution is presented in Table 1. Simulation computed tomography (CT) scans were acquired with patients in the supine position on a breast board, with the left arm elevated above the head. Imaging was performed during the inspiratory phase under deep DIBH, with a slice thickness of 3 mm.

**TABLE 1:** Patient distribution by treatment region, cohort, and fractionation schedule.

	Breast only	Breast with PLN	Total	25fr x2Gy	15fr x2.67
KB model	13	32	45	42	3
Validation	0	20	20	16	4
Post‐implementation	10	5	15	5	10
Total	23	57	80	63	17

Clinical target volumes (CTVs) were determined based on tumor stage, surgical intervention, and nodal involvement. Planning target volumes (PTVs) were generated by applying a 5‐mm isotropic margin to the CTV, while the external body contour was contracted by 3 mm to define the optimization boundary. Contouring followed ESTRO guidance and was adapted individually for each patient.^19^ Anatomically diverse cases were intentionally included to support model robustness and generalizability.

### 2.2 Planning and delivery (DIBH/SGRT)

**FIGURE 1:**
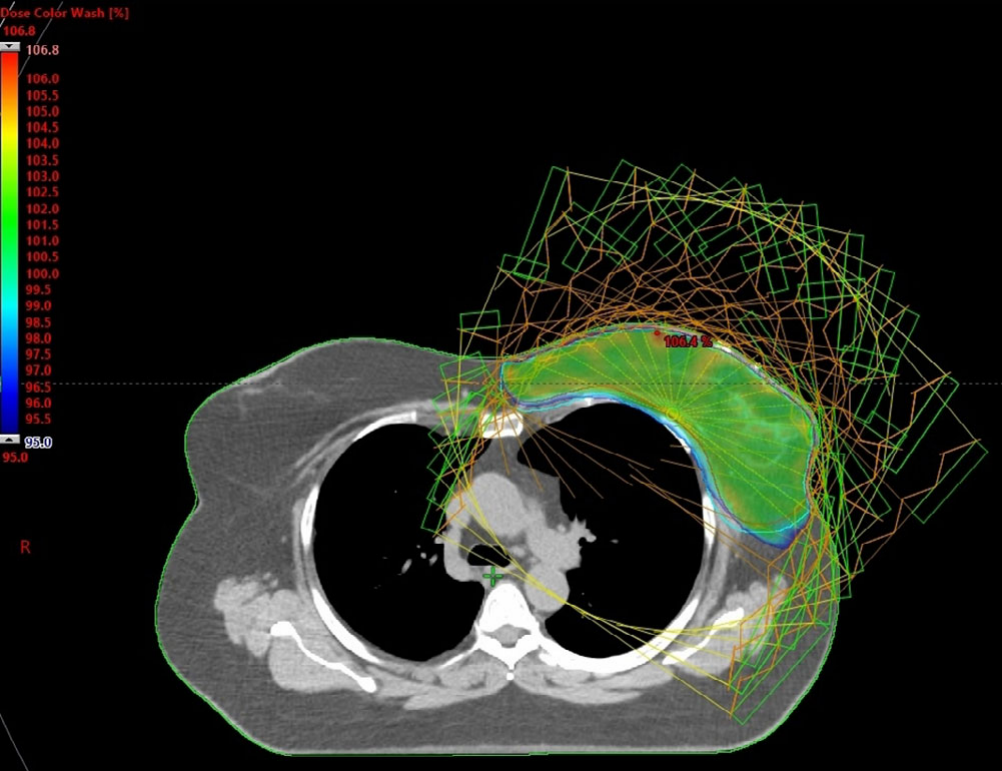
Fixed beam configuration geometry used for IMRT planning across all cases.

Radiotherapy was delivered on the Ethos™/Halcyon platform (v1.0, Varian Medical Systems, Palo Alto, USA) O‐ring linear accelerator operated in nonadaptive mode. Treatment plans were generated in the Eclipse Treatment Planning System (v16.1, Varian Medical Systems, Palo Alto, USA) for delivery on the Halcyon platform using 6‐MV FFF beams. An 18‐field IMRT technique was employed, with beam angles selected to avoid direct lung entry. The beam configuration geometry is illustrated in Figure 1. Beam configurations, gantry angles, collimator settings, and energy parameters were held constant across all cases. Treatment plans were generated using a standardized institutional planning template incorporating predefined DVH‐based optimization objectives. The training dataset consisted exclusively of clinically approved plans created using this template, ensuring consistent objective definition across cases. Manual iterative optimization was performed to achieve institutional target coverage and OAR constraints. Plans were optimized using the Photon Optimizer and calculated with the Acuros XB algorithm. A surface‐guided radiation therapy system (AlignRT InBore™, Vision RT Ltd., London, UK) was used to monitor and track patients during DIBH.

OARs included the heart, LAD, spinal canal, contralateral breast (R_Breast), ipsilateral lung (L_Lung), and contralateral lung (R_Lung). Auxiliary optimization structures included Body–PTV (a ring region 4 cm beyond the PTV within the body contour), healthy lung (lung tissue ≥3 cm from the PTV), a 1.5–3 cm peritumoral ring, and a medial dummy volume in the upper slices adjacent to the heart representing vascular structures, used to aid medial dose reduction. All normal‐tissue contours were generated using ART‐Plan (Therapanacea, v2.3.1) and reviewed by a dosimetrist. Normal Tissue Objective (NTO) values were set based on clinical experience.

### 2.3 KBP development and evaluation

All included patients had completed radiotherapy and had clinically approved IMRT plans. Outlier identification and management were performed using regression and residual plots generated by RapidPlan™ engine (Varian Medical Systems, Palo Alto, USA) for each anatomical structure. From the initial 60 candidate plans considered for model development, 15 plans were excluded following outlier analysis and clinical review, resulting in a final training dataset of 45 plans. The KBP model was ultimately trained on 45 high‐quality plans involving patients receiving radiotherapy for breast only and for breast with peripheral lymph nodes. Outlier detection was performed using the model analytics tools integrated in the Eclipse KBP module. Regression and residual plots generated for each anatomical structure were evaluated together with statistical indicators including Cook's distance (>10), studentized residuals (>3), modified *Z*‐score (mZ >3.5), and geometric outlier flags as described in previous literature. Plans flagged during statistical screening underwent individual clinical review and, when appropriate, re‐optimization was attempted. Cases demonstrating persistent non‐representative dosimetric characteristics were excluded from the training dataset.

The excluded outlier cases were retained within the overall study cohort and incorporated into the independent validation dataset in order to evaluate model performance in anatomically or dosimetrically atypical cases and to assess model generalizability.Model performance was summarized using the coefficient of determination (*R* ^2^) and chi‐square (*X* ^2^) statistics for targets and OARs, while mean squared error (MSE) was used to assess predictive accuracy. Model validation was conducted in an independent cohort of 20 patients not included in training. Clinical feasibility was assessed in 15 additional patients via comparative planning, generating three plans per case:

1. Clinical Plan (ClinP): the clinically delivered plan.
2. KBP Plan: a model‐only plan generated from KBP DVH estimates with no manual optimization.
3. KBP+MP Plan: a manually refined version of the KBP plan to evaluate potential improvement via limited planner intervention.

KBP results were first assessed without manual edits; KBP + MP was then applied to determine whether parameters not meeting desired values could be improved with targeted iterations.

For the KBP cohort, after importing model‐predicted DVH objectives, a single optimization was performed without further manual modification of constraints, weights, or beam geometry. In contrast, the KBP + MP cohort involved limited manual refinement consisting of selective OAR objective weight or upper limit adjustments, while keeping beam configuration and target objectives unchanged.

The PTV was defined as breast tissue and regional lymphatics cropped 3 mm beneath the skin surface. For inverse‐planned IMRT, our institutional planning objective was PTV_Dmax ≤ 112% of the prescription dose, while the clinical acceptability threshold was defined as PTV_Dmax ≤ 115%, provided that hotspots were confined within the PTV and all OAR constraints were satisfied.

Dosimetric comparisons across the three plan types used DVH endpoints, including PTV_D98, PTV_D95%, PTV_D2%, PTV_Dmax, homogeneity index (PTV_HI), and conformity index (PTV_CI) and OAR endpoints (Heart_Dmean, Heart_D2%, LAD_Dmean, LAD_D2%, R_Breast_Dmean/D2%, L_Lung_Dmean, L_Lung_V5%/V20%/D2%, R_Lung_Dmean/ D2%/ V5%, Spinal Canal). Total monitor units (MUs) were also recorded.

### 2.4 Statistics and QA

For the 15‐patient feasibility cohort, all three plans were measured using the ArcCHECK phantom system (Sun Nuclear Corporation, USA) on the Ethos/Halcyon‐equivalent platform. Gamma pass rates were analyzed using the 3%/3‐mm criterion. Dosimetric results for ClinP, KBP, and KBP+MP were compared using the paired *t* test and the Wilcoxon signed‐rank test. All statistical analyses were performed in IBM SPSS Statistics version 23 (SPSS Inc., Chicago, IL, USA).

## 3 RESULTS

### 3.1 KBP model performance

The KBP model was developed using RapidPlan to generate clinically acceptable dose‐volume predictions. Its statistical performance was evaluated based on *R* ^2^ and *X* ^2^, which respectively reflect the strength of the geometric‐dosimetric correlation and the agreement between predicted and actual DVH outcomes.

The *R* ^2^ value demonstrated high predictive accuracy for structures: R_Lung (*R* ^2^ = 0.943) showed excellent correlation between anatomical geometry and dosimetric outcomes, indicating highly reliable model performance in the structures. Good predictive correlation was also observed for the whole lung (*R* ^2^ = 0.742), L_Lung (*R* ^2^ = 0.650), Heart (*R* ^2^ = 0.602), LAD (*R* ^2^ = 0.459), and spinal canal (*R* ^2^ = 0.546), reflecting acceptable model behavior for major thoracic OARs. In contrast, the lowest *R* ^2^ value was recorded for R_Breast (*R* ^2^ = 0.163), suggesting higher variability or less geometric dependence of the dose in this region.

The chi‐square (*X* ^2^) values ranged from 1.007 to 1.120 across all structures, supporting good agreement between predicted and achieved doses. The LAD exhibited a *X* ^2^ value of 1.058, consistent with clinically acceptable prediction accuracy. The heart (*X* ^2^ = 1.110), L_Lung (*X* ^2^ = 1.120), and spinal canal (*X* ^2^ = 1.084) also showed acceptable levels of agreement. The lowest *X* ^2^ values were observed for the R_Breast (*X* ^2^ = 1.007) and R_Lung (*X* ^2^ = 1.008), reflecting high model precision in these cases despite the lower R^2^ for the R_Breast. *R* ^2^, *X* ^2^, and MSE results of OARs are presented in Table 2.

**TABLE 2:** *R* ^2^, *X* ^2^, and MSE values demonstrating KBP model performance.

Structure	*R* ^2^	*χ* ^2^	MSE
Heart	0.602	1.110	0.02
LAD	0.459	1.058	0.02
L_Lung	0.650	1.120	0.04
R_Lung	0.943	1.008	0.00
Total lungs	0.742	1.089	0.02
R_Breast	0.163	1.007	0.02
Spinal canal	0.546	1.084	0.02

Abbreviations: MSE, mean squared error; *R* ^2^, coefficient of determination; *X* ^2^, chi‐square statistic.

### 3.2 Target coverage, dose homogeneity, and conformity index

The KBP and KBP + MP plans were compared with the previously clinically validated ClinP and the results were as follows: the mean ± standard deviation (SD) of PTV_D98 was 94.0 ± 1.46% for the Clinical Plan, 94.3 ± 1.52% for the KBP plan and 94.5 ± 1.49% for the KBP + MP plan generated by continuous manual iteration. PTV_D95 values were similar in the ClinP and KBP plans (97.2 ± 0.89% and 97.2 ± 1.15%, respectively), whereas a slight decrease was observed in the KBP + MP plan (97.0 ± 0.95%). No statistically significant differences were found among the ClinP, KBP, and KBP + MP plans for either PTV_D98 or PTV_D95 (*p* > 0.05). These findings indicate that model‐based planning achieved a level of target volume coverage comparable to that of clinically approved plans.

PTV_Dmax was 108.6 ± 1.9% in the ClinP and showed a slight increase in the KBP and KBP + MP plans, reaching 111.3 ± 2.24% and 110.9 ± 2.2%, respectively. This increase was statistically significant; however, it remained within our predefined institutional acceptability threshold (PTV Dmax ≤ 115%).

**FIGURE 2:**
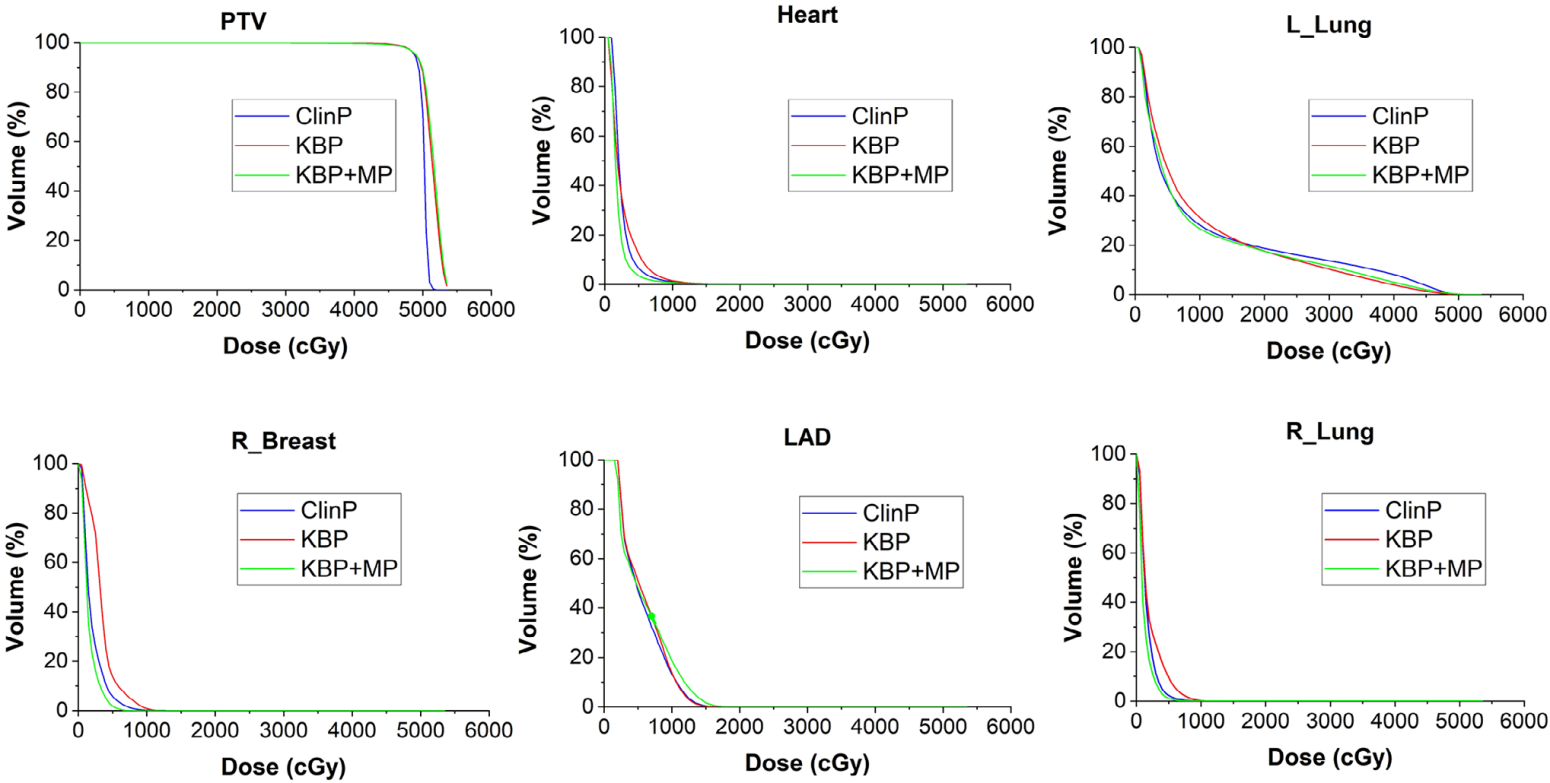
Cumulative DVH comparison of ClinP, KBP, and KBP + MP plans for the PTV and OARs.

**FIGURE 3:**
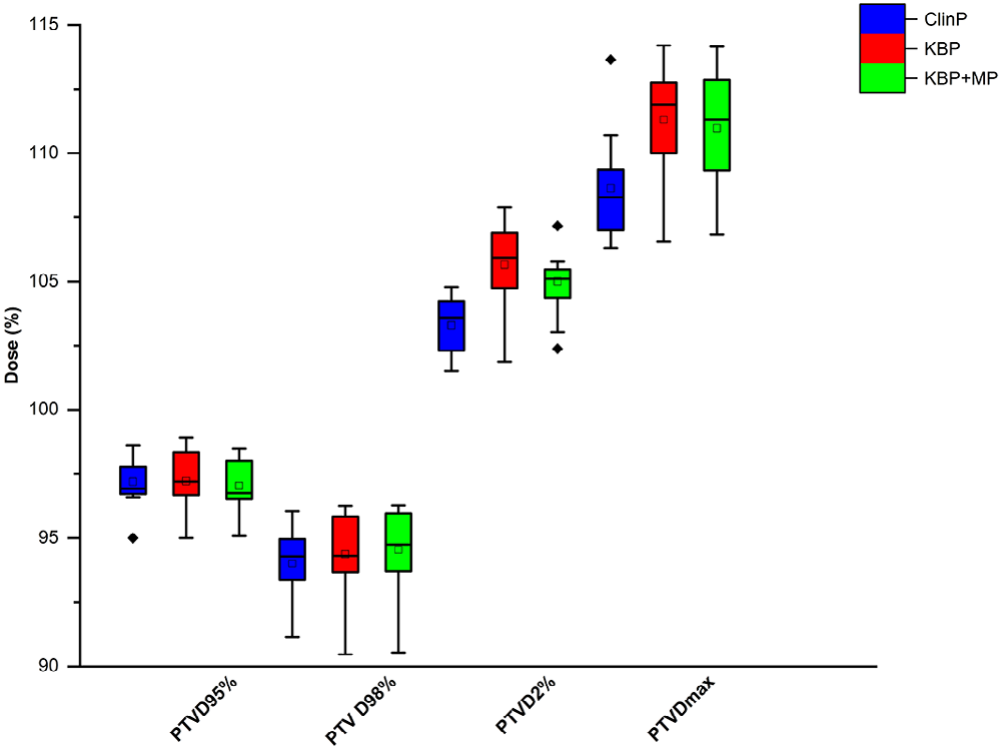
Box plots of PTV dose parameters across ClinP, KBP, and KBP + MP plans.

The cumulative DVH comparison for the ClinP, KBP, and KBP + MP plans across the PTV and selected OARs is shown in Figure 2. The figure visually illustrates the dose distribution differences among the three planning approaches. Figure 3 presents comparative box plots of PTV_ D95%, D98%, D2%, and Dmax values for ClinP, KBP, and knowledge‐based plus manual intervention plans.

**FIGURE 4:**
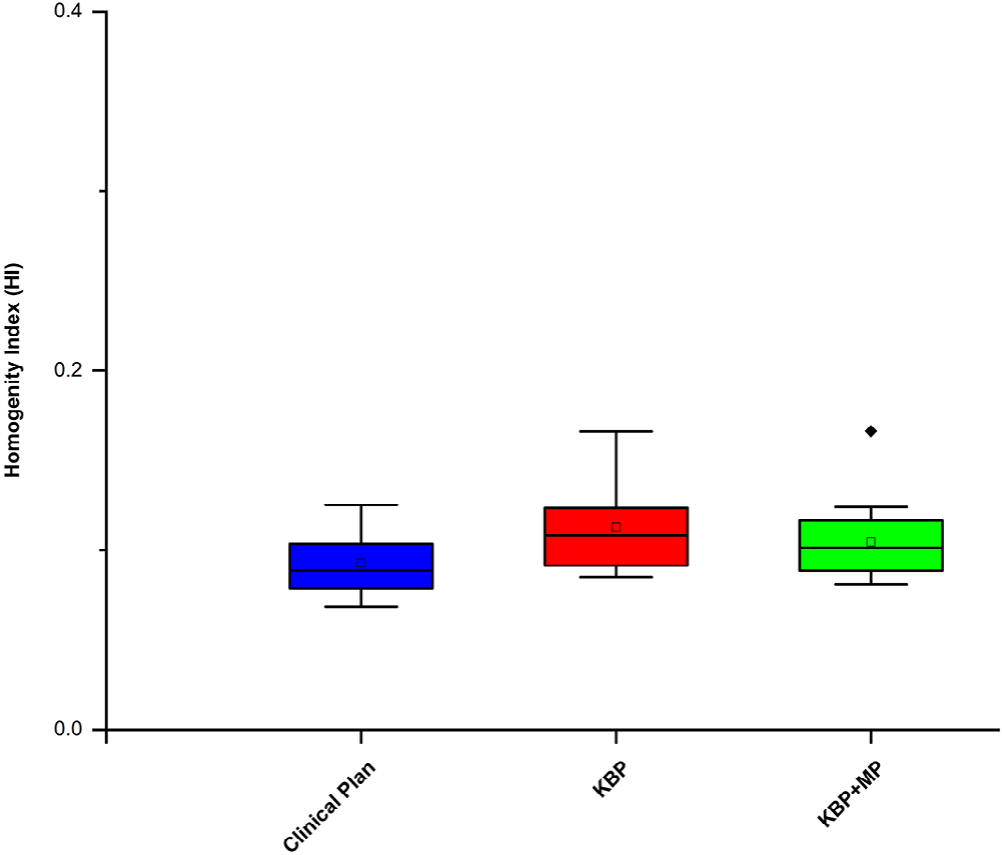
Comparison of the homogeneity index among ClinP, KBP, and KBP + MP plans.

The mean ± SD Homogeneity Index (HI) was 0.090 ± 0.02 for the ClinP, 0.113 ± 0.022 for the KBP plan, and 0.105 ± 0.022 for the KBP+MP plan. Statistical analysis revealed that the ClinPs were significantly more homogeneous than the KBP plans (*p* = 0.019). While KBP + MP improved homogeneity compared to KBP alone, it did not reach the level observed in the ClinPs, as illustrated in Figure 4.

**FIGURE 5:**
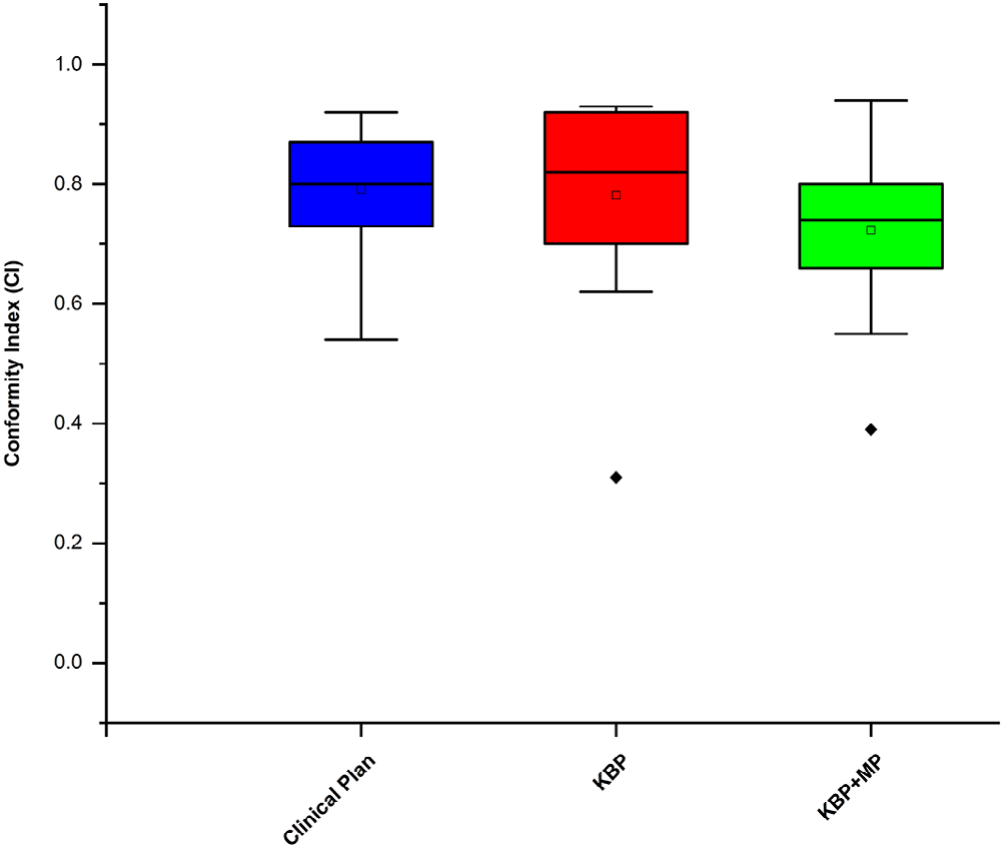
Comparison of the conformity index among ClinP, KBP, and KBP + MP plans.

The Conformity Index (CI) was similar in the ClinP (0.79) and KBP(0.78), whereas a slight decrease was observed with KBP + MP (0.70). As shown in Figure 5, Statistical analysis showed no significant differences in CI among the plans.

### 3.3 OAR dose comparisons

Heart_Dmean was significantly higher in the KBP (*p* = 0.001), whereas no significant difference was observed between KBP + MP and the ClinP (*p* = 0.088). This finding suggests that KBP tends to increase heart dose; however, this effect can be partially corrected through manual adjustments.

No significant differences were found among the plans in terms of Heart_D2%. However, the significant difference observed between KBP and KBP + MP (*p* = 0.008) indicates that high doses can be reduced through manual adjustment.

LAD_ Dmean doses were found to be similar across all plans (*p* = 0.112, *p* = 0.156). LAD_ D2% was significantly lower in both KBP and KBP + MP plans compared with the ClinPs, with *p*‐values of 0.041 and 0.047, respectively.

Compared with the ClinP, R_Breast_Dmean dose was significantly higher in the KBP plans (*p* = 0.002). This increase was found to be reducible with KBP + MP (*p* = 0.100). L_Lung D2% was significantly lower in the KBP plans (*p *= 0.028), whereas in the KBP + MP plans the result was similar to that of the ClinP (*p* = 0.084).

**FIGURE 6:**
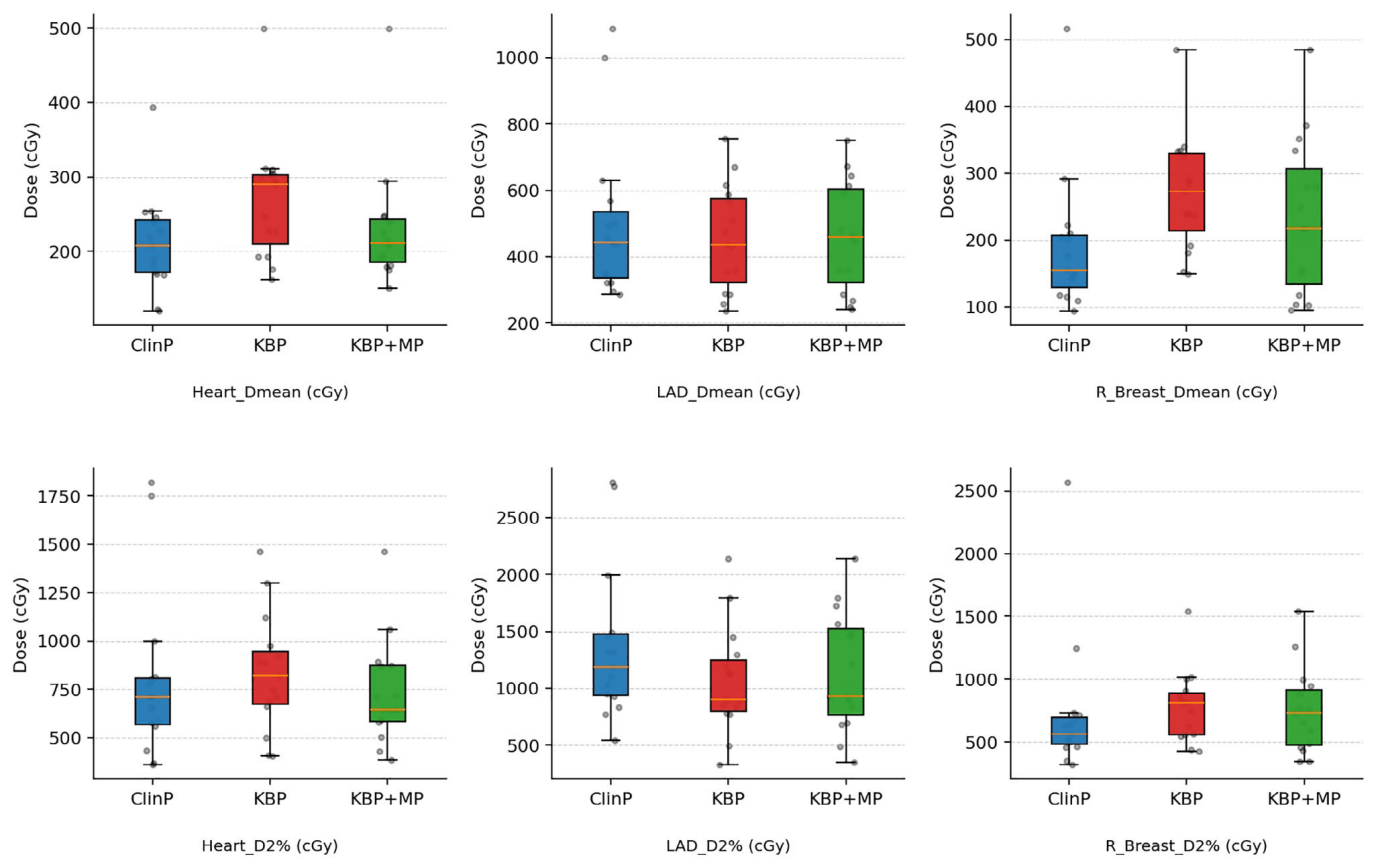
Comparison of Dmean and D2% for the heart, LAD, and contralateral breast among ClinP, KBP, and KBP + MP plans.

As shown in Figure 6, the KBP + MP approach resulted in lower mean heart and contralateral breast doses compared to KBP alone, while maintaining comparable LAD dose parameters to the clinical plans.

**FIGURE 7:**
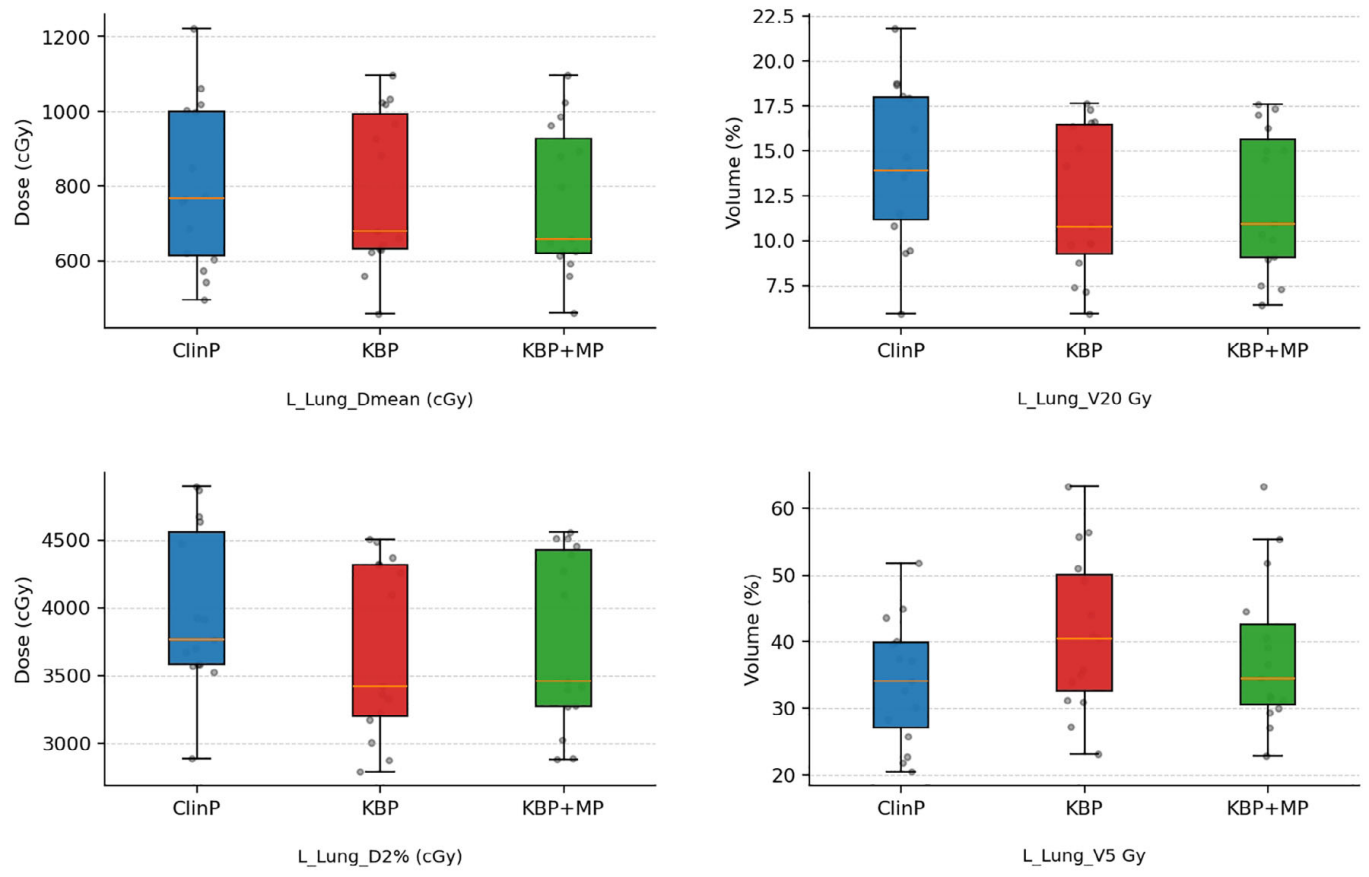
Comparison of Dmean, D2%, V20, and V5 for the ipsilateral lung among ClinP, KBP, and KBP + MP plans.

L_Lung V5Gy was found to be higher in both KBP and KBP + MP plans, with the differences being statistically significant *p* = 0.001 and *p* = 0.031, respectively. This finding reflects a broader low‐dose spread in KBP, which was reduced with KBP + MP. In contrast, V20% was significantly lower in both KBP and KBP + MP plans, with *p*‐values of 0.004 and 0.002, respectively. L_Lung Dmean doses were similar between the ClinP and KBP, but were found to be significantly lower in the KBP + MP plans (*p* = 0.004). As presented in Figure 7, KBP resulted in a lower ipsilateral lung V20 compared to ClinP, while the V5 value was higher. KBP + MP achieved an intermediate profile, reducing V5 compared to KBP while maintaining a similar V20.

**FIGURE 8:**
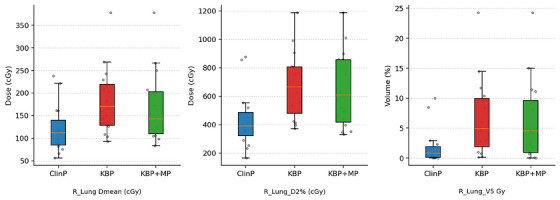
Comparison of Dmean, D2%, and V5 for the contralateral lung among ClinP, KBP, and KBP + MP plans.

R_Lung D2%, V5%, and Dmean were significantly increased in the KBP plans (*p* < 0.01). Although these increases were reduced with KBP + MP, they remained significantly higher compared with the Clinical Plan (*p* < 0.05). These findings suggest that KBP alone may be insufficient for sparing the right lung and that manual intervention is necessary. As shown in Figure 8, KBP resulted in higher mean and near‐maximum doses to the contralateral lung compared to ClinP, while KBP + MP slightly reduced these values but did not achieve the lower levels observed in the clinical plans.

### 3.4 Monitor units, tables, and quality assurance

Total MU values were similar between the ClinP and both KBP and KBP + MP, with *p*‐values of 0.776 and 0.118, respectively. However, KBP + MP, which involved additional iterations, required significantly higher MU compared with single‐iteration KBP plans (*p* = 0.001). The mean total MU for the ClinP was 2125 ± 508, whereas it was 3% higher for KBP (2190 ± 366) and 11% higher for KBP + MP (2359 ± 405).

The mean ± SD (min–max) values and *p*‐values from the statistical comparisons are presented in Table 3 for PTV and in Table 4 for OARs.

**TABLE 3:** Comparison of PTV DVH parameters among ClinP, KBP, and KBP + MP plans.

Parameter	ClinP Mean ± SD (min‐max)	KBP Mean ± SD (min‐max)	KBP + MP Mean ± SD (min‐max)	ClinP versus KBP *p*‐value	ClinP versus KBP + MP *p*‐value	KBP versus KBP + MP *p*‐value
PTV_D98% (%)	94.0 ± 1.46(91.1–96.1)	94.3 ± 1.52(90.5–96.3)	94.5 ± 1.49(90.5–96.3)	0.426	0.226	0.068
PTV_D95% (%)	97.2 ± 0.89 (95.0–98.6)	97.2 ± 1.15(95.0–98.92)	97.0 ± 0.95 (95.1–98.48)	0.930	0.551	0.144
PTV_Dmax (%)	108.6 ± 1.9 (106.3–113.7)	111.3 ± 2.24 (106.6–114.2)	110.9 ± 2.2 (106.8–114.2)	0.001	0.001	0.418
PTV_HI	0.09 ± 0.02 (0.07–0.125)	0.11 ± 0.02 (0.09–0.17)	0.11 ± 0.02 (0.08–0.17)	0.019	0.102	0.017
PTV_CI	0.79 ± 0.09 (0.54–0.92)	0.78 ± 0.17 (0.31–0.93)	0.72 ± 0.13 (0.39–0.94)	0.839	0.083	0.031

Abbreviations: CI, conformity index.;ClinP, clinically accepted plan; HI, homogeneity index; KBP, knowledge‐based planning; KBP+MP, knowledge‐based planning with additional manual iteration; PTV_D2%, dose received by 2% of the PTV; PTV_D95%, percentage of the prescription dose received by 95% of the PTV; PTV_D98%, dose received by 98% of the PTV.

**TABLE 4:** Comparison of OAR DVH parameters among ClinP, KBP, and KBP + MP plans.

Parameter	ClinP Mean ± SD (min‐max)	KBP Mean ± SD (min‐max)	KBP + MP Mean ± SD (min‐max)	ClinP versus KBP *p*‐value	ClinP versus KBP + MP *p*‐value	KBP versus KBP + MP *p*‐value
Heart Dmean (Gy)	2.11 ± 0.67(1.20–3.94)	2.69 ± 0.83(1.62–4.99)	2.31 ± 0.83(1.50–5.00)	**0.001**	0.088	**0.001**
Heart_D2% (Gy)	7.94 ± 4.38 (3.59–18.21)	8.33 ± 3.02(4.05–14.61)	7.34 ± 2.72 (3.85–14.62)	0.334	0.532	**0.008**
LAD_Dmean(Gy)	5.09 ± 2.39 (2.87–10.89)	4.55 ± 1.60(2.36–7.56)	4.59 ± 1.66(2.42–7.51)	0.112	0.156	0.589
LAD_D2%(Gy)	13.68 ± 6.76 (5.42–28.10)	10.66 ± 4.71(3.27–21.41)	11.45 ± 5.25(3.48–21.38)	**0.041**	**0.047**	**0.001**
R_Breast Dmean (Gy)	1.90 ± 1.04 (0.94–5.17)	2.70 ± 0.88(1.49–4.84)	2.34 ± 1.17(0.96–4.84)	**0.002**	0.100	0.061
R_Breast D2% (Gy)	7.28 ± 5.53 (3.19–25.66)	7.83 ± 2.84 (4.29–15.40)	7.48 ± 3.40 (3.47–15.41)	0.211	0.532	0.363
L_Lung D2%(Gy)	39.77 ± 5.91(28.86–48.94)	37.01 ± 6.42(27.91–45.02)	37.59 ± 6.51(28.81–45.57)	**0.028**	0.084	**< 0.001**
L_Lung V5Gy(%)	34.1 ± 9.2(20.57–51.77)	41.2 ± 11.8(23.11–63.23)	38.7 ± 11.4(22.90–63.27)	**0.001**	**0.031**	**0.001**
L_Lung V20Gy (%)	14.3 ± 4.35 (5.92–21.78)	12.2 ± 4.2 (5.91–17.62)	12.2 ± 4.0 (6.39–17.58)	**0.004**	**0.002**	0.570
L_Lung Dmean(Gy)	7.99 ± 2.19 (4.97–12.21)	7.90 ± 2.09 (4.59–10.98)	7.63 ± 1.98 (4.61–10.98)	0.532	0.078	0.004
R_Lung D2%(Gy)	4.32 ± 2.04(1.65–8.75)	6.81 ± 2.40(3.72–11.87)	6.46 ± 2.69(3.31–11.88)	**0.001**	**0.001**	0.078
R_Lung V5Gy(%)	1.9 ± 3.1(0–9.98)	6.8 ± 6.6(0.13–24.24)	6 ± 6.9(0.02–24.27)	**0.001**	**0.004**	**0.033**
R_Lung Dmean (Gy)	1.20 ± 0.54(0.56–2.38)	1.83 ± 0.76(0.92–3.78)	1.68 ± 0.81(0.84–3.78)	**0.001**	**0.003**	**0.003**
Total MU	2125 ± 508(1347–2802)	2190 ± 366(1679–3191)	2359 ± 405(1736–3322)	0.776	0.118	**0.001**

**Abbreviations**: ClinP, clinically accepted plan; KBP, knowledge‐based planning; KBP+MP, knowledge‐based planning with additional manual iteration; SD, standard deviation.

Based on the dosimetric comparison of DVH parameters for OARs, KBP plans achieved values closest to the ideal for LAD_Dmean, LAD_D2%, L_Lung D2%, L_Lung V20, and L_Lung Dmean when compared with both ClinP and KBP + MP.

In contrast, KBP + MP plans yielded values closest to the ideal for Heart_D2% and L_Lung Dmean compared with Clinical and KBP plans. When manual optimization was applied to KBP plans, Heart_Dmean, Heart_D2%, R_Breast Dmean, R_Breast D2%, L_Lung V5, L_Lung Dmean, R_Lung D2%, R_Lung V5, and R_Lung Dmean met the predefined dose constraints relative to KBP alone. While KBP plans were able to reduce doses for certain OARs such as the LAD and left lung, they tended to increase doses to other organs, including the heart, contralateral breast, and left lung. KBP + MP plans were found to substantially mitigate the disadvantages introduced by KBP.

For quality assurance, all ClinP, KBP and KBP + MP plans of 15 patients were measured using the ArcCHECK phantom on the Halcyon platform. Using the 3%/3 mm criteria, gamma pass rates were found to be above 97%.

## 4 DISCUSSION

This study aimed to evaluate the ability of a KBP model to generate clinically acceptable treatment plans for left‐sided breast cancer patients treated with DIBH technique. The performance of the KBP model was assessed across different clinical scenarios. Despite the heterogeneity of the patient cohort, the *R* ^2^, *X* ^2^, and MSE values indicated that the model achieved sufficient predictive capability to be considered of moderate accuracy. However, low *R* ^2^ and high MSE values observed for certain OARs suggest that additional manual optimization may be necessary to further improve the model's performance.

Comparison of ClinP with KBP and KBP + MP plans revealed no statistically significant differences in the target volume parameters PTV_D98, PTV_D95, and CI. This finding indicates that model‐based planning achieved a level of target volume coverage comparable to that of clinical plans.

Previous studies have reported that mean heart doses achieved with KBP were lower compared with manual plans.^20^, ^21^, ^22^ The moderate *R* ^2^ and MSE values obtained for the heart indicate that the model is statistically adequate for predicting heart doses; however, manual input may still be required. Compared with clinical plans, KBP plans showed a significant increase in mean heart dose. Although the DIBH technique aims to displace the heart away from the PTV, in left‐sided breast radiotherapy the anatomical proximity of the heart to the target volume and individual anatomical variations may lead to discrepancies between the predicted DVH outputs and clinical expectations. It is considered necessary for heart doses to be carefully reviewed by the planner, with dose constraints checked to determine the need for additional manual optimization.

For the ipsilateral lung, V_20 and D_2% values were significantly reduced in both KBP and KBP + MP plans compared with the clinical plans, whereas V5 values were higher in both plan types. This indicates that while KBP is effective in limiting the high‐dose volume of the lung, it may have certain limitations in preventing low‐dose spread. Similar findings have been reported in the literature. Esposito PG et al. observed superior ipsilateral Lung_V5 and V20 values in KBP plans,^22^ while Wang et al. reported that KBP plans achieved better ipsilateral lung sparing, particularly for V20, compared with manual plans.^20^ In contrast, Fogliata et al. found a tendency for ipsilateral lung doses to increase in KBP plans.^23^

Previous studies have reported varying results regarding the effectiveness of KBP in reducing contralateral breast and lung doses. While some studies found KBP to be superior,^20^, ^24^, ^25^ others consistent with our findings reported that manually generated clinical plans achieved significantly better sparing in regions distant from the target volume, such as the contralateral lung and contralateral breast.^21^ The marked increase observed in contralateral Lung_V5 dose may be attributed to insufficiently defined constraints for low‐dose regions during model training or to a lack of prioritization for contralateral organs during the plan optimization process.

Our results support the clinical applicability of the model while indicating that further optimization or clinical adjustments may be required in certain cases. Although KBP models have the potential to reduce high‐dose regions compared with clinical plans, they may increase low‐dose spread, with more pronounced increases observed in distant OARs such as the contralateral lung and contralateral breast. In our study, the KBP + MP approach enabled more balanced and optimized control of OAR doses.

Although manual optimization following KBP increases the overall planning and delivery time, this extension is considered acceptable in light of the potential benefits it provides in terms of OAR sparing. While KBP applications in breast radiotherapy have been previously reported, most studies have focused on VMAT techniques or isolated dosimetric comparisons. The present study extends existing literature by providing a structured IMRT‐based DIBH evaluation incorporating post‐implementation assessment and planner–model interaction analysis. These findings offer practical guidance for standardized KBP integration into routine clinical workflows and highlight clinically relevant trade‐offs in low‐dose distribution.

The validation cohort included anatomically diverse cases, including those excluded from model training during outlier screening, allowing assessment of model robustness in nonideal anatomical situations. In this context, the contribution of the present study lies not in proposing a new optimization algorithm but in demonstrating the practical implementation, reproducibility, and clinical integration of KBP within a standardized DIBH breast IMRT workflow.

In the present study, the analysis focused on IMRT‐based DIBH planning; however, VMAT has also become widely adopted in breast radiotherapy. The KBP framework is, in principle, adaptable to different delivery techniques; however, VMAT‐specific characteristics such as arc geometry, modulation complexity, and low‐dose spread behavior would necessitate independent model training and validation. In principle, VMAT arc geometries could be combined with model‐estimated DVH objectives to generate VMAT‐based plans; however, such an approach was not formally evaluated in the present study. Therefore, the current findings cannot be directly extrapolated to VMAT without technique‐specific model development. Future investigations may explore structured KBP implementation within VMAT‐based DIBH workflows.

## 5 CONCLUSION

This study demonstrates the feasibility of implementing a structured knowledge‐based IMRT workflow for left‐sided breast radiotherapy under DIBH conditions. The trained model achieved consistent target coverage and OAR sparing within predefined institutional acceptability criteria. In the KBP arm, plan generation was completed following a single model‐driven optimization without additional manual constraint adjustment. Compared with conventional planning, the KBP approach reduced the extent of user‐dependent iterative refinement while maintaining dosimetric quality. These findings support the integration of structured KBP workflows into standardized DIBH breast IMRT planning.

## AUTHOR CONTRIBUTIONS

Oznur Senkesen: Participated in the study design, data collection, performed statistical analysis, and contributed to manuscript writing, Emine Burcin Ispir: Contributed to statistical analysis and treatment planning, Irem Aydin: Contributed to data collection and treatment planning, Evrim Tezcanli: Performed clinical evaluation of the treatment plans.

All authors read and approved the final manuscript and agreed to be accountable for all aspects of the work

## FUNDING INFORMATION

This research did not receive any specific grant from funding agencies in the public, commercial, or not‐for‐profit sectors.

## CONFLICT OF INTEREST STATEMENT

The authors have no conflict of interest.

## ETHICS STATEMENT

This study was approved by the Acibadem Mehmet Ali Aydınlar University Ethics Committee (approval no. ATADEK‐2025/15).

## Data Availability

The data that support the findings of this study are available from the corresponding author upon reasonable request, subject to institutional and ethical approvals.
